# Spatial Study of TLR4, TLR5 and TLR9 in Gastric Premalignant Lesions Before and After *Helicobacter pylori* Eradication

**DOI:** 10.3390/ijms26094059

**Published:** 2025-04-25

**Authors:** Franz Villarroel-Espíndola, Leyla Jaupi, Joaquín Reyes, Carlos Barrientos, Celia Podestá, Carolina Selman, Carolina Bizama, Alejandro Corvalan, Roxana Gonzalez-Stegmaier, Sergio Jara-Rosales, Pia Bascur

**Affiliations:** 1 Translational Medicine Unit, Fundación Arturo López Pérez (FALP) Cancer Center, Santiago 7500921, Chile; leyla.jaupi@falp.org (L.J.); joaquin.reyes@falp.org (J.R.); roxana.gonzalez@falp.org (R.G.-S.); sergio.jara@falp.org (S.J.-R.); pia.bascour@falp.org (P.B.); 2 Advanced Center for Chronic Diseases (ACCDIS), Santiago 8331150, Chile; cbizamas@uc.cl (C.B.); acorvalan@uc.cl (A.C.); 3 PhD Program in Chronic Diseases, Faculty of Medicine and Science, Universidad San Sebastián, Los Leones Campus, Santiago 7510157, Chile; 4 PhD Program in Medical Sciences, Faculty of Medicine, Universidad Austral de Chile, Valdivia 5090000, Chile; 5 Endoscopy Unit, Fundación Arturo López Pérez (FALP) Cancer Center, Santiago 7500921, Chile; barrientosc@falp.org (C.B.); celia.podesta@falp.org (C.P.); 6 Molecular Diagnosis Laboratory, Diagnostic Units, Fundación Arturo López Pérez (FALP) Cancer Center, Santiago 7500921, Chile; carolina.selman@falp.org; 7 Department of Pathology, UC-Center for Investigational Oncology (CITO), School of Medicine, Pontificia Universidad Católica de Chile, Santiago 8331150, Chile; 8 Faculty of Health Care Sciences, School of Midwifery, Universidad San Sebastián, Los Leones Campus, Santiago 7510157, Chile

**Keywords:** Toll-like receptor, gastric cancer, operative link on gastritis assessment, *Helicobacter pylori*

## Abstract

The histological changes in the gastric epithelium are crucial in the progression from premalignant to neoplastic lesions. TLR4, TLR5 and TLR9 have been localized in the gastric epithelium and studied separately using conventional histological techniques without a focus on the protein or cell interactions within the microenvironment. Therefore, we developed a multiplex immunohistochemistry/immunofluorescence (mIHC/IF) technology for the simultaneous detection of TLR4, TLR5 and TLR9 on a single tissue section of human gastric biopsies from 10 paired cases collected in two independent visits, and its correlation with the OLGA/OLGIM scoring and *H. pylori* status after eradication. The results confirmed that mIHC/IF is useful for simultaneously interrogating six biomarkers and demonstrated that TLR4 and TLR9 are significantly associated with *H. pylori* infection. However, only TLR9 is positively related to the presence of intestinal metaplasia. TLR5 was mainly present in goblet cells (TFF3+) but did not show any significant association with *H. pylori* or the presence of intestinal metaplasia. Our results suggest that a more comprehensive strategy to interrogate the tissue microenvironment in premalignant lesions may improve the interpretation of the earned risk of gastric cancer in patients with chronic gastritis and evidence of failure in *H. pylori* eradication.

## 1. Introduction

Multiomic approaches based on DNA and RNA have provided large datasets for explorations of tumor characteristics as well as the prognostic significance of the tumor microenvironment (TME) [1]; however, those technologies lack spatial information. Conventional immunohistochemistry (IHC) is a widely used diagnostic technique in tissue pathology, and it can distinguish between different cell types expressing the same protein and can characterize the density and spatial distribution of specific cells within the TME [2,3]. IHC can also provide a semi-quantitative assessment of marker intensity. On the other hand, immunofluorescence (IF) has shown the benefit of being able to characterize a large dynamic range of signal detection, in addition to the localization and spatial distribution of the positive cells. Multiplex immunohistochemistry/immunofluorescence (mIHC/IF) technologies, which allow the simultaneous detection of multiple markers on a single tissue section, have been introduced and adopted in both research and clinical settings in response to the increased demand for improved techniques [2,3].

The tumor microenvironment (TME) represents a complex interaction between elements of the host and tumor cells, including a variety of immune cells and stromal cells (blood vessels and fibroblasts), each one characterized by specific protein expression patterns and representing a potentially unique niche [1]; however, the properties of the premalignant tissue microenvironment is still poorly understood.

According to the Correa model, intestinal-type gastric cancer (GC) is preceded by premalignant lesions, including chronic atrophic gastritis (CAG) and intestinal metaplasia (IM) [4]. The histological changes in the gastric epithelium are crucial in the progression of the cascade from premalignant lesions to the malignant lesion. For example, CAG is characterized as the loss of parietal cells and pepsinogen-producing chief cells, while IM is defined as the emergence of intestinal-specific cell types, including goblet cells and enterocytes [5,6]. Previously, the molecular features of gastric cancer and premalignant lesions were explored, indicating that GAG exhibits a pronounced mitochondrial gene expression signature associated with *Helicobacter pylori* (*H. pylori*, Hp) infection, and for the intestinal metaplasia the molecular profile was associated with increased expression of intestinal differentiation genes, which were not overexpressed in tumor cells [7,8].

In addition, low and high grades of intraepithelial neoplasia have shown similar expression profiles compared to early GC [9], characterized by an increase in the stemness of gastric epithelial cells, with at least 27 consistent genes contributing to the cell transformation, and an immune microenvironment more active in the early cancer than in premalignant lesions, especially in the infiltration of lymphocytes and macrophages [9].

Gastric epithelial cells represent the first line of innate immunity of the stomach and the first defense against *H. pylori*, responding to infection by numerous cell signaling cascades, including cytokine induction and the subsequent recruitment of inflammatory cells to the gastric mucosa [4,5]. Toll-like receptor (TLR) family members mediate many of inflammatory events and these are expressed by the gastric epithelium under normal conditions, as well as modulated by the presence of pathogens or neoplasia [10,11].

TLR4, TLR5 and TLR9 have been histologically localized in patients with non-inflamed gastric mucosa and in Hp-related gastritis [12,13], and an upregulation of TLR2 and TLR4 has been reported in the gastric antral region more than in the gastric body; this may be influenced by the stimulatory effect of the colonization of *H. pylori* [14], and these changes in TLR abundance can be also observed during the progressive transformation from metaplasia to dysplasia [15]; however, in advanced malignant lesions, the TLR expression seems to be diminished [16]. On the other hand, the presence of TLR2, TLR4 and TLR9 has also been described in infiltrating immune cells, mainly monocytes/macrophages across the Correa’s cascade [17]. Recently, the circulating levels of soluble forms of TLR2, TLR4, and TLR9 were positively associated with advanced stages of gastric cancer, including an upregulation of the protein levels on circulating immune cells in patients with gastric cancer compared to those with premalignant lesions [18].

Unfortunately, most of the data reported above have been collected separately using single staining procedures, considering that mIHC/IF provides high-throughput multiplex staining and standardized quantitative analysis for highly reproducible, efficient and cost-effective tissue studies. This is the first study analyzing simultaneously the expression pattern of TLR4, TLR5 and TLR9 in human gastric biopsies from paired cases collected in two independent visits separated by 3 years, and correlating with the *H. pylori* status and histological assessment of atrophy and intestinal metaplasia by Operative Link for Gastritis Assessment (OLGA) and Operative Link on Gastric Intestinal Metaplasia (OLGIM) scoring. Our results allow us to suggest a more comprehensive strategy to interrogate the tissue microenvironment in premalignant lesions and evaluate the earned risk of gastric cancer in patients with chronic gastritis and evidence of failure in the *H. pylori* eradication.

## 2. Results

### 2.1. Clinical Intervention Promotes Regression of the Atrophic Status but Does Not Reduce Risk of H. pylori Infection

**Figure 1:**
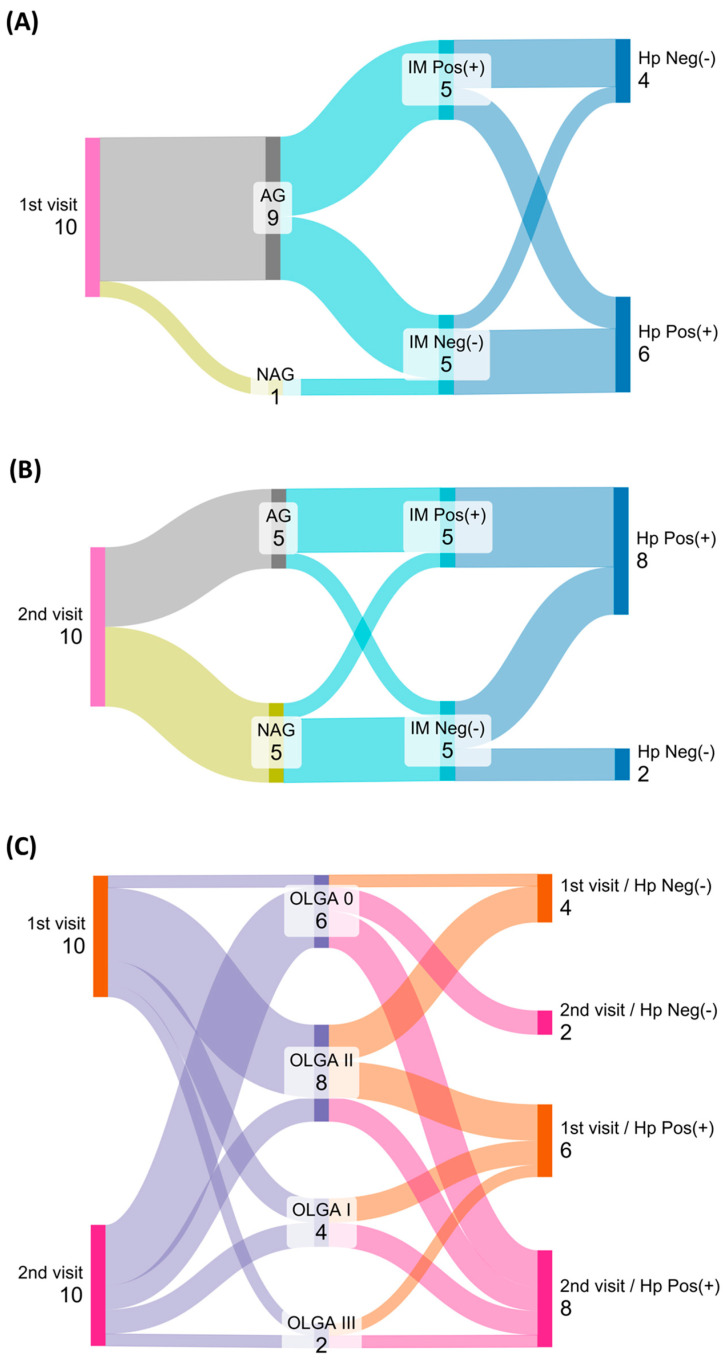
Medical intervention promotes regression of the atrophic status but does not reduce risk of *H. pylori* infection. Clinical information from 10 paired cases collected after two separate visits for endoscopic assessment. Sankey diagram was used to represent histological classifications and status of *H. pylori* infection during the first (1st) and the second (2nd) visit (**A**,**B**). The overall segregation based on OLGA scoring and response after *H. pylori* eradication was summarized (**C**). Hp: *Helicobacter pylori*; Neg(−): negative; Pos(+): positive; NAG: non-atrophic gastritis; AG: atrophic gastritis; IM: intestinal metaplasia.

Understanding the clinical relevance of a comprehensive tissue analysis of endoscopic biopsies from asymptomatic individuals to reveal the effects of a clinical intervention to reduce risk of gastric cancer, we analyzed 20 paired samples collected endoscopically during two independent visits, each one separated by 3 years. Even though during the first visit the participants did not report previous symptoms or evidence of gastric issues (Table 1), 9 out of 10 cases showed signs of atrophic gastritis, including 6 out of 10 that were positive for *H*. *pylori* based on ELISA test and Giemsa staining. On the other hand, during the second visit and after a gastrointestinal intervention, the number of gastric atrophies was 5 out of 10, with 8 positive cases for *H. pylori*. In both visits, the proportion of cases with intestinal metaplasia did not show differences. Based on a Sankey’s diagram (Figure 1A,B), the evolution of cases between the first and second visit suggests that the medical interventions might prevent the progression of the atrophic process, and when the cases were scored based on OLGA the redistribution of cases showed a significant movement from stage II to stage O/I; however, the presence of *H*. *pylori* infection was not modified, suggesting a failure of the antibiotic therapy or re-infections.

### 2.2. Histological Premalignant Progression Requires a Persistent Infection of H. pylori

**Figure 2:**
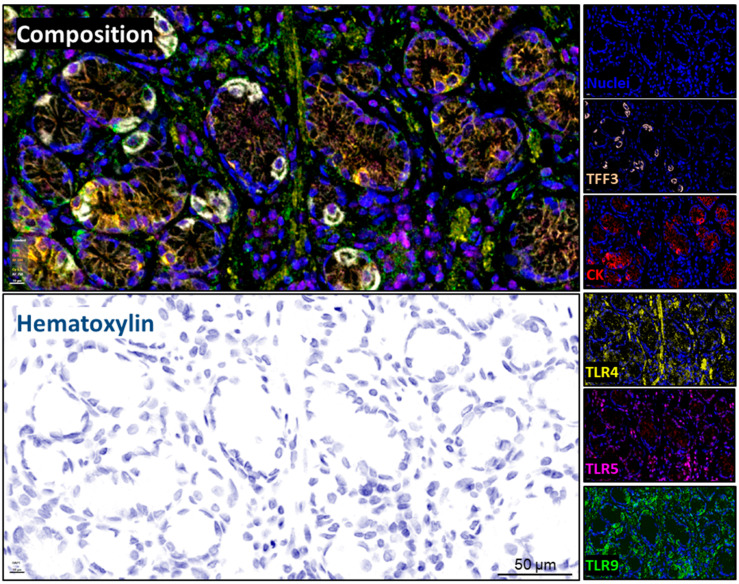
Multiplexed immunohistochemistry/immunofluorescence (mIHC/IF) panel. Representative images showing each biomarker as separated channels (insets) and the combined composition using the indicated pseudo-coloring code. The pseudo-hematoxylin staining was built in Phenochart using DAPI signal. Nuclei were co-stained with DAPI. TFF3: trefoil factor 3; CK: cytokeratin; TLR: Toll-like receptor. Original capture 20×, scale bar: 10 µm, digital magnification 40× bar: 50 µm.

**Figure 3:**
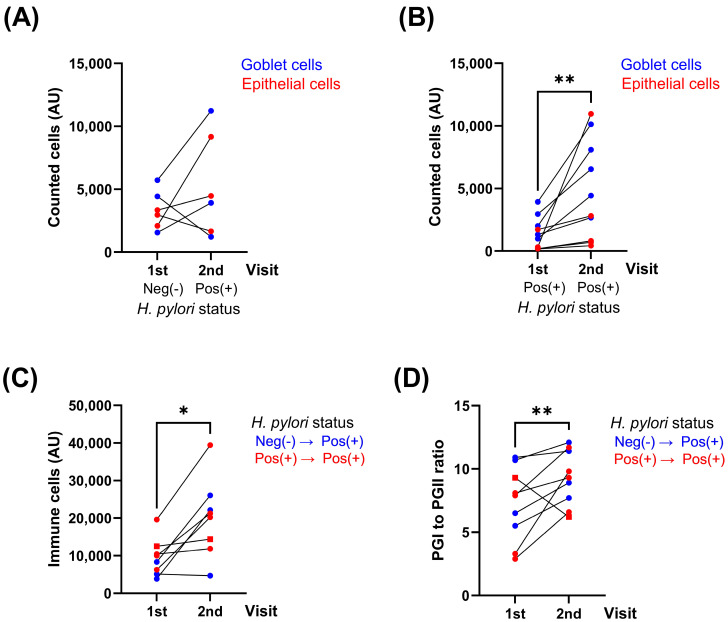
Progression of premalignant lesions requires a persistent infection with *H. pylori*. Changes between the first (1st) and second (2nd) visit were represented using connected line graph. Total counted cells were obtained after tissue segmentation and cell phenotyping. Goblet and epithelial cells were counted as TFF3 positive and CK positive (TFF negative) objects in cases segregated according to *H. pylori* presence in each visit (**A**,**B**). The changes in the total number of infiltrating immune cells (any object TLR positive, TFF3 and CK negative) were compared between visits (**C**). Serum levels of pepsinogen I (PGI) and pepsinogen II (PGII) were measured by ELISA (GastroPanel^®^, Biohit) and compared (**D**). Pos(+): positive; Neg(−): negative. A Wilcoxon matched-pairs test was applied, * *p* < 0.01; ** *p* < 0.001.

Having in consideration our previous results, we developed the simultaneous immunodetection of three members of the Toll-like receptor family (TLR4, TLR5 and TLR9), including protein markers for goblet and epithelial cells, TFF3 and cytokeratin, respectively. The staining pattern for each biomarker was as expected (Figure 2), showing a cytoplasmic–membranous distribution, including a very specific mutual exclusion localization for TFF3 and cytokeratin, allowing the individualization of goblet (TFF3+) and epithelial cells (TFF3−/CK+) and the segregation of cases with mild and severe signs of intestinal metaplasia or atrophy, respectively, using the mean fluorescent intensity of each marker. On the other hand, TLR4 was widely detected in the gastric epithelium, as well as TLR9, which also displayed high intensity on goblet cells (insets). Regarding TLR5, it showed an intracellular distribution and was frequently observed in mononuclear cells infiltrating the stroma of the gastric tissue. More representative images are presented in Supplementary Data (Figure S1), showing the variability in the intensity of the fluorescent signal and the distribution of the different targets between non-related tissue sections. After tissue segmentation and cell phenotyping, each paired case was compared between the first and second visit based on its *H. pylori* status (Figure 3), showing that cases initially negative and later positive for *H. pylori* did not display significant differences in the counted number of goblet (TFF3+) or epithelial cells (TFF3−/CK+) per tissue section (normalized by area and squared pixels) (Figure 3A). However, in those cases where the eradication of *H. pylori* was not satisfactory, a significant increase in the number of goblet cells was observed (Figure 3B). In addition, the presence of *H. pylori* during the second visit was characterized by an increased abundance of infiltrating immune cells within the stromal compartment of the gastric epithelium and an elevated pepsinogen I to pepsinogen II ratio in serum (Figure 3C and Figure 3D, respectively).

### 2.3. Toll-like Receptors Are Selectively Overexpressed in Atrophic or Metaplastic Conditions

**Figure 4:**
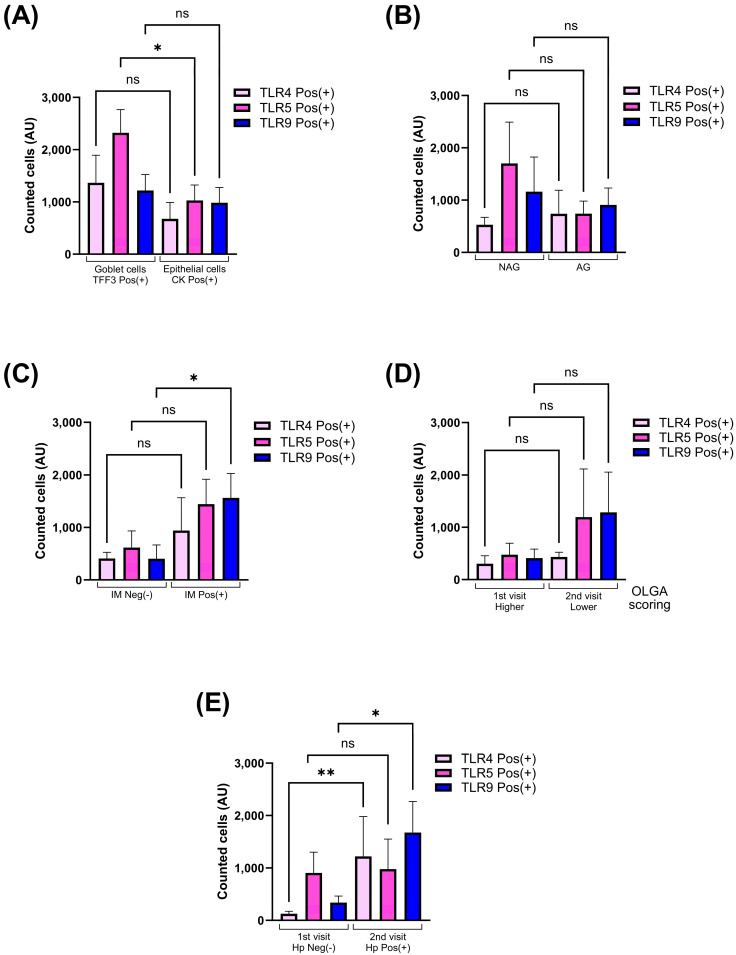
Toll-like receptors are selectively overexpressed in atrophic and metaplastic conditions. Changes and distribution in the total cells counted as positive for TLR4, TLR5 and TLR9 are represented as bar graph as mean ± SEM. TLR expression in goblet and epithelial cells is compared (**A**). Number of cells expressing TLRs is compared in atrophic and metaplastic conditions (**B**,**C**). Number of positive cells for TLR4, TLR5 or TLR9 are compared based on the OLGA scoring during each visit (**D**), or based on the *H. pylori* status after a medical intervention (**E**). Hp: *Helicobacter pylori*; Neg(−): negative; Pos(+): positive; NAG: non-atrophic gastritis; AG: atrophic gastritis; IM: intestinal metaplasia. Kruskal–Wallis and Dunn’s multiple comparisons test were applied, ns: non-significant; * *p* < 0.01; ** *p* < 0.001. Bonferroni’s correction was performed after the Dunn’s test.

TLR4, TLR5 and TLR9 were widely present in all cells of the gastric epithelium; however, the number of cells double positive for TLR5 and TFF3 was significantly higher than any other phenotype (Figure 4A). Interestingly, none of the three receptors showed a significant change between non-atrophic and atrophic tissue; however, the number of positive cells for TLR9 was significantly elevated (*p*-value < 0.01) in cases with intestinal metaplasia (Figure 4B and Figure 4C, respectively). In addition, all cases were grouped according to the severity of the atrophic gastritis using their respective OLGA scoring as lower (I–II) and higher (III–IV), and this segregation did not show differences in the number of cells expressing any of the three Toll-like receptors (Figure 4D). On the other hand, *H. pylori* infection itself was able to demonstrate a significant effect on the expression of TLR4 and TLR9, showing a significant increased number of cells expressing TLR4 (*p*-value < 0.001) and TLR9 (*p*-value < 0.01), without changes in TLR5 (Figure 4E).

## 3. Discussion

Multiplex immunofluorescence (mIF) provides the scope to examine interactions between the tumor and the immune compartment at a single-cell resolution using a panel of antibodies that can be chosen based on the type of cancer or the clinical interest of the study, but it can also provide meaningful data in premalignant lesions and early stages of epithelial neoplasia. With high throughput imaging platforms, researchers can develop spatial modeling methods to evaluate the risk of histological progression and the effectiveness of therapeutic procedures, as many other researchers have suggested [19]. To our knowledge, we have reported the first approach to interrogate in a multiplexed manner the presence of Toll-like receptors within the context of atrophy and intestinal metaplasia of human FFPE samples. In general, the presence of some polymorphisms of the TLR4 gene might be associated with a higher risk of gastric cancer [20,21]. TLR5 is frequently related with the recognition of *H. pylori* flagellin [22], but it seems that this bacterium has developed mechanisms to escape this recognition representing an important factor involved in the persistence of this infection. TLR9 is involved in the recognition of *H. pylori* DNA but is the only TLR with an anti- and pro-inflammatory action and it could be able to promote or suppress the infection with *H. pylori* according to the gastric environment [23,24].

Although we considered a very small sample group for this work, we had at least three biological replicates per condition, and our results were consistent with other reports using classic immunohistochemistry and molecular assays [13,14]. Using confocal microscopy, TLR4, TLR5 and TLR9 have been observed in the antrum and corpus of the stomach samples from patients with *H. pylori*-related gastritis and with non-inflamed gastric mucosa [13]. Previously, in patients with clear evidence of peptic ulcers, *H. pylori* infection showed a significant increase in TLR2 and TLR4 mRNA expression in the antral region compared to uninfected samples [14]. At the subcellular level, TLR4 was localized at the apical and the basolateral domain of the gastric epithelium, independently of the presence or absence of *H. pylori*. However, TLR5 and TLR9, in the presence of *H. pylori,* changed its location from the apical portion to an exclusive basolateral localization [13], suggesting that the sentinel role of these receptors may be dynamically regulated by a polarized distribution on the surface of the epithelial cells. Unfortunately, the relationship between TLR and *H. pylori*-related gastric diseases and its progression is still controversial [25]. Recently, the tissue expressions of TLR1, TLR2, TLR4, TLR5, TLR7 and TLR9 as potential prognostic biomarkers in gastric cancer were reported, showing a positive correlation between each other and a high expression of each studied TLR in tumors with an intestinal-type histology [26]. However, in another study, the expression of TLR1, TLR2, TLR4, TLR5 and TLR6 was associated with survival in gastric cancer, and only high cytoplasmic TLR2 expression was shown to be significantly associated with a poorer 5-year survival [27].

Our results align with the findings reported by Schmausser et al. (2004), who described for the first time the presence of TLR4, TLR5 and TLR9 in inflamed and non-inflamed gastric tissue, including its localization within the epithelium with a sign of acute and chronic inflammation promoted by the presence of *H. pylori* [13]. In addition, our results agreed with the most recent observations reported by Ding et al. (2022), indicating that high levels of TLR9 are associated with the *H. pylori*-infected stomach and correlated with metaplasia and cancer [28]. However, our findings are discordant according to the work published by Schmausser et al. (2005), who reported that TLR9 was not detectable in intestinal metaplasia or dysplasia [12]. Compared with the previous work, the authors modified the TLR9 staining protocol adding an incubation step with proteinase K; probably, it affected the antigen detection causing an irregular staining pattern. However, the protein abundance and expression pattern of TLR4 and TLR9 could be associated with and influenced by polymorphisms, which have been previously associated with chronic *H. pylori* infection in the Indian population [29]. Therefore, it would be valuable to have a subsequent follow up of these patients who present metaplasia associated with *H. pylori* to explore in future studies whether the expression of TLR9 or the presence of reported polymorphism predisposes them to subsequent neoplastic complications.

Many authors have suggested the participation of TLR during the cancer onset and the tumor progression, and, eventually, these have been related to the prognosis of different tumor types [30], limiting their observations to associations between TLR expression and histological changes. Even though we have reported significant associations between TLR9 and TLR5 in metaplastic tissue, our research needs the exploration of plausible molecular explanations for our results. The published experimental data in gastric tissue are mainly limited to *H. pylori* recognition; however, from the immunological evidence, TLR activation can launch cascades leading, for instance, to the induction of pro-inflammatory cytokines, promotion of angiogenesis, and stimulation of cell migration, which are recognized hallmarks of cancer [31], so the study of these downstream intracellular pathways might be a good starting point for further investigations.

We recognize the limitations of our study, mainly the small number of cases studied; however, considering the strong reliability reported for the multiplexed imaging tools, our results are still meaningful to set up larger further studies and implement predictive models including multiple biomarkers. Most of the published studies in our country have considered a very limited number of participants and short periods of follow up, according to the experimental design or the characteristic of the measured endpoint. For this work, we only reported a follow up of three years for 10 paired cases, which is not enough to fully observe the malignant transformation of the gastric mucosa; according to Yamada et al. (2025), a follow up of at least 4 years is required to find a primary gastric tumor (27 out of 1757 participants) [32].

In addition, we understand that the exploration of clinical outcomes and biological endpoints, such as the progression of premalignant lesions to cancer cells, might be limited by the number of participants or the number of observed events, depending also on a high acceptance rate or a low drop-off rate during the study. Regarding two comparable experimental approaches, these limitations must be considered for further studies. Based on the IARC worldwide *H. pylori* prevalence survey (ENIGMA) applied in Chile, the estimated rates of acceptance to participate in that study varied between 8.6 and 33.3% [33]. Previously, Arenas et al. (2019) described from a cohort of 121 patients that only 57% completed and finished the study [34].

Regarding gastric cancer screening, the current strategy in Chile consists of an upper gastrointestinal endoscopy for people aged 40 years or more with epigastric pain. Local published data indicate that, between 2009 and 2010, the endoscopy coverage in this objective population was 14.4% [35]. However, the risk of tissue damage progression caused by an infection with *H. pylori* is still poorly managed, including the assessment of antibiotic susceptibility. A contemporary study reported that OLGA scores, atrophy, metaplasia and GC increased significantly with age; however, *H. pylori* infection was more frequent in people below 57 years old [36]. The Chilean clinical guideline for the eradication of *H. pylori* in patients with peptic ulcer was published in 2013 [37]. This guideline indicates as a first-line treatment the combined use of a standard dose of a proton-pump inhibitor (PPI), 500 mg of clarithromycin and 1 gr of amoxicillin each 12 h for 14 days. As second line, in addition to the PPI, it will include 120 mg of bismuth subsalicylate, 250 mg of metronidazole and 500 mg of tetracycline each 6 h for 7 to 14 days. The guideline only suggests the confirmation of a successful eradication using a non-invasive method, but it is not encouraged as routine. Within the metropolitan region of Chile, the reported presence of antibiotic-resistant *H. pylori* strains was 20% for clarithromycin, for tetracycline 26.8% and for metronidazole 44.9% [38]. More recently, the prevalence of levofloxacin and clarithromycin resistance in Santiago city was 29% and 27%, respectively [39]. In fact, a local cohort study of 121 enrolled patients with a positive urease test showed a *H. pylori* eradication rate of 63% with a prevalence of clarithromycin resistance was 26% [34]. This information is consistent with our data, and may explain the observed rate of H. pylori positivity during the second visit.

In summary, most of the previously published data on Chilean people have used serological and epidemiological approaches to control the success of the therapies to eradicate *H. pylori* [40,41,42], leaving an open field to explore the evolution of the gastric epithelium. Currently several clinical studies are evaluating the frequency of H. pylori infection in Chilean children and adolescents and the effectivity of the antibiotic regimen in this population [43,44,45], representing a very early onset of gastric disease with an outcome that will be influenced by social and environmental factors.

Our approach for tissue analysis will contribute to a better characterization of the premalignant lesions after bacterial eradication and their impact on the earned risk of gastric cancer in Chilean adults with concomitant risk factors, such as obesity or diagnosed metabolic diseases. Therefore, this work may represent a starting point to complement, in a comprehensive manner, the histological changes and the effectiveness of clinical interventions to reduce gastric cancer statistics in our population.

## 4. Materials and Methods

### 4.1. Endoscopic Procedure

All patients presented with 12 h fasting and were administered intravenous sedation with midazolam (0.05 mg per kg of weight) according to the Chilean Ministry of Health guidelines for this procedure. All endoscopies were performed using an Evis EXERA III gastro-scope (GIF-1TH190, Olympus, Santiago, Chile). Tech-Bite™ Biopsy forceps (Micro-tech Endoscopy, Ann Arbor, MI, USA) were used to collect five gastric mucosa samples according to the Sydney Protocol for the Operative Link for Gastritis Assessment (OLGA). Biopsy specimens were fixed in 10% Neutral Phosphate Buffered Formalin (Leica, Deer Park, IL, USA).

### 4.2. FFPE Samples and Case Selection

Cross-sectional and retrospective samples from 10 paired patients that underwent an upper endoscopic assessment during 2016 and 2019 were included according to the protocol 2021-021-RES-GAS-FIN approved by the Institutional Ethical Committee of Fundacion Arturo Lopez Perez Cancer Center (July 2021). Briefly, the original protocol considered men and women without symptoms of gastric illness within the 6 months previous to the enrollment; everyone signed a consent letter to participate in a longitudinal study with 3 years’ follow up and agreed two endoscopic assessments at the Fundacion Arturo Lopez Perez Cancer Center in 2016 and 2019. The original study had as aims to evaluate effectivity of *H. pylori* eradication, serum levels of IgG against *H. pylori* and other molecular biomarkers using liquid biopsy, according to the clinical protocol N° 14-280 and N° OLGA-2 approved by the Ethical Committees of Pontifical Catholic University of Chile (July 2015) and by the Occident Medical Service of Santiago (August 2019). Historical formalin-fixed and paraffin-embedded (FFPE) blocks were collected from the internal biorepository; 20 FFPE cassettes were selected and paired with the respective patient and procedure. Clinical information and anatomical pathology reports were identified for the two visits (2016 and 2019). All available tissue biopsies were examined by a pathologist to confirm the histology and the integrity of the material using hematoxylin–eosin and Giemsa staining.

### 4.3. Multiplexed Immunofluorescence

Immunostaining was carried out as published [46,47]. One multiplexed panel was developed for this study, using the following monoclonal antibodies: anti-TLR4 (clone 76B357-1, Novus Biologicals, Centennial, CO, USA), anti-TLR5 (clone 19D759.2, Novus Biologicals, Centennial, CO, USA), anti-TLR9 (clone 26C593.2, Novus Biologicals, Centennial, CO, USA), anti-TFF3 (clone EPR3974, Abcam, Cambridge, UK) and anti-cytokeratin (clone AE1/AE3, Dako, Agilent, Santa Clara, CA, USA). Briefly, after antigen retrieval, each primary antibody was incubated 30 min at room temperature and sequentially detected using the respective host-specific HRP-conjugated secondary antibody. Tyramide-based amplification systems were used for signal detection according to the manufacturer’s instructions and assigned specifically for each biomarker (Supplementary Table S1). After co-staining with DAPI, each slide was cover slipped in Prolong Gold mounting medium (Invitrogen, ThermoFischer Sci., Waltham, MA, USA). An index TMA was stained in parallel as quality control. This array included human tissue as naturally positive and negative controls, such as skeletal muscle (negative and noise threshold), tonsil, colon and placenta. The ratio between the average fluorescent signals from a positive control above the mean noise (muscle) was used for each antibody as a reference for visual positivity in the samples of interest.

### 4.4. Slide Scanning and Image Digitization

All slides were scanned at 20× magnification using a PhenoImager^®^ HT 2.0 microscope (Akoya Biosciences, Marlborough, MA, USA). Exposure times were determined for each target using an index TMA including normal human tissue as positive and negative controls, and the respective auto-fluorescence slide, according to the instructions of the provider. The optimal exposure time considered the optimal signal-to-noise ratio measured for each target, and the supervised observation of the operator for the visual positive signal and the expected staining pattern. Later, an unsupervised, automatic and simultaneous scanning was applied to each whole tissue section.

### 4.5. Analysis for Single Objects

Whole-slide scans from the fluorescent-labeled sections were analyzed using Phenochart workflow for spectral unmixing, viewing and annotating digital slides, and inForm^®^ v.3.0.0 software for the automated tissue analysis and comparative studies. In addition, for some targets, a manual verification was performed using QuPath v.0.3.2 software. Briefly, DAPI signal was used for cell segmentation, and at least 40 cells from different cores were used to train each object classifier, as recommended by the provider. Data were exported to an Excel file and used for further analysis.

### 4.6. Statistical Analysis

All analyses were performed using GraphPad Prism 8.0 (GraphPad Software, San Diego, CA, USA). Data normality was assessed for each group prior to selecting the appropriate statistical test. Depending on the distribution, comparisons between two groups were conducted using either the Student’s *t*-test or the Mann–Whitney U test, while comparisons among multiple groups were analyzed using parametric ANOVA. Graphs display the mean ± standard error of the mean (SEM). Statistical significance was defined as *p* < 0.05, * *p* < 0.01 and ** *p* < 0.001. Diagram for patients across visits was created using SankeyMATIC.

## 5. Conclusions

This work represents a comprehensive approach to study the histological changes and the effectiveness of clinical interventions to reduce gastric cancer risk in the Chilean population using a multiplex immunohistochemistry/immunofluorescence (mIHC/IF) technology and the simultaneous detection of TLR4, TLR5 and TLR9 on formalin-fixed and paraffin-embedded tissue sections of gastric epithelium obtained by endoscopic biopsy.

## Figures and Tables

**Table 1:** Clinical data and demographic information of studied cases.

CASES = 10		First Visit							Second Visit						
ID Case	Sex	Age	AG*	IM	PGI	PGII	Ratio	Hp	Age	AG*	IM	PGI	PGII	Ratio	Hp
CBB2012	Male	71	II	Yes	142.2	13.0	10.9	N	74	III	Yes	209.4	18.3	11.4	P
CBB2007	Female	56	II	No	57.5	19.5	2.9	P	59	II	Yes	106.2	16.1	6.6	P
CBB2023	Male	55	II	Yes	47.0	8.6	5.5	N	58	O	Yes	62.7	8.1	7.7	P
CBB2006	Female	66	O	No	52.7	10.6	5.0	N	70	O	No	54.6	7.9	6.9	N
CBB2010	Male	58	II	Yes	56.5	5.3	10.7	N	62	II	Yes	58.3	5.0	11.7	P
CBB2051	Female	55	I	No	66.4	10.2	6.5	P	59	O	No	46.2	5.2	8.9	N
CBB2054	Male	68	II	No	141.7	17.4	8.1	P	71	O	No	113.5	12.2	9.3	P
CBB2014	Female	73	I	No	434.1	55.2	7.9	P	76	I	No	165.6	14.2	11.7	P
CBB2058	Female	63	II	Yes	98.9	1.06	9.3	P	66	II	Yes	43.3	7.0	6.2	P
CBB2025	Male	55	III	Yes	188.0	56.8	3.3	P	58	I	Yes	124.4	12.7	9.8	P
CBB2053	Male	52	O	No	1.0	9.2	0.1	N	55	I	No	4.6	6.3	0.7	N

Participants agreed to two endoscopic assessments separated by three years, including intervention with antibiotic for *H. pylori* infection. Age (years); AG*: OLGA scoring for atrophic gastritis; IM: intestinal metaplasia, all positive cases showing isolated focus of mild metaplasia; PGI: serum pepsinogen I (µg/L); PGII: serum pepsinogen II (µg/L); Ratio: PGI to PGII ratio; Hp: *H. pylori* status by Giemsa and PCR; P: positive; N: negative.

## Data Availability

The data that support the findings of this study are available on request from the corresponding author [F.V.-E.]. The data are not publicly available due to ethical restrictions.

## Supplementary Materials

The following supporting information can be downloaded at: https://www.mdpi.com/article/10.3390/ijms26094059/s1.

## Abbreviations

The following abbreviations are used in this manuscript:

TLR	Toll-like receptor
OLGA	Operative Link on Gastritis Assessment
