# Supplementary material for: Spatial Study of TLR4, TLR5 and TLR9 in Gastric Premalignant Lesions Before and After Helicobacter pylori Eradication
Source: Int J Mol Sci. 2025 Apr 25;26(9):4059. doi: 10.3390/ijms26094059 (PMC12072049; doi:10.3390/ijms26094059)
Supplement: Supplementary file 1 [file ijms-26-04059-s001.zip › ijms-3590905-supplementary.pdf]

Supplementary Data

Spatial study of TLR4, TLR5 and TLR9 in gastric premalignant lesions before and after *Helicobacter pylori* eradication

Franz Villarroel-Espíndola et al.

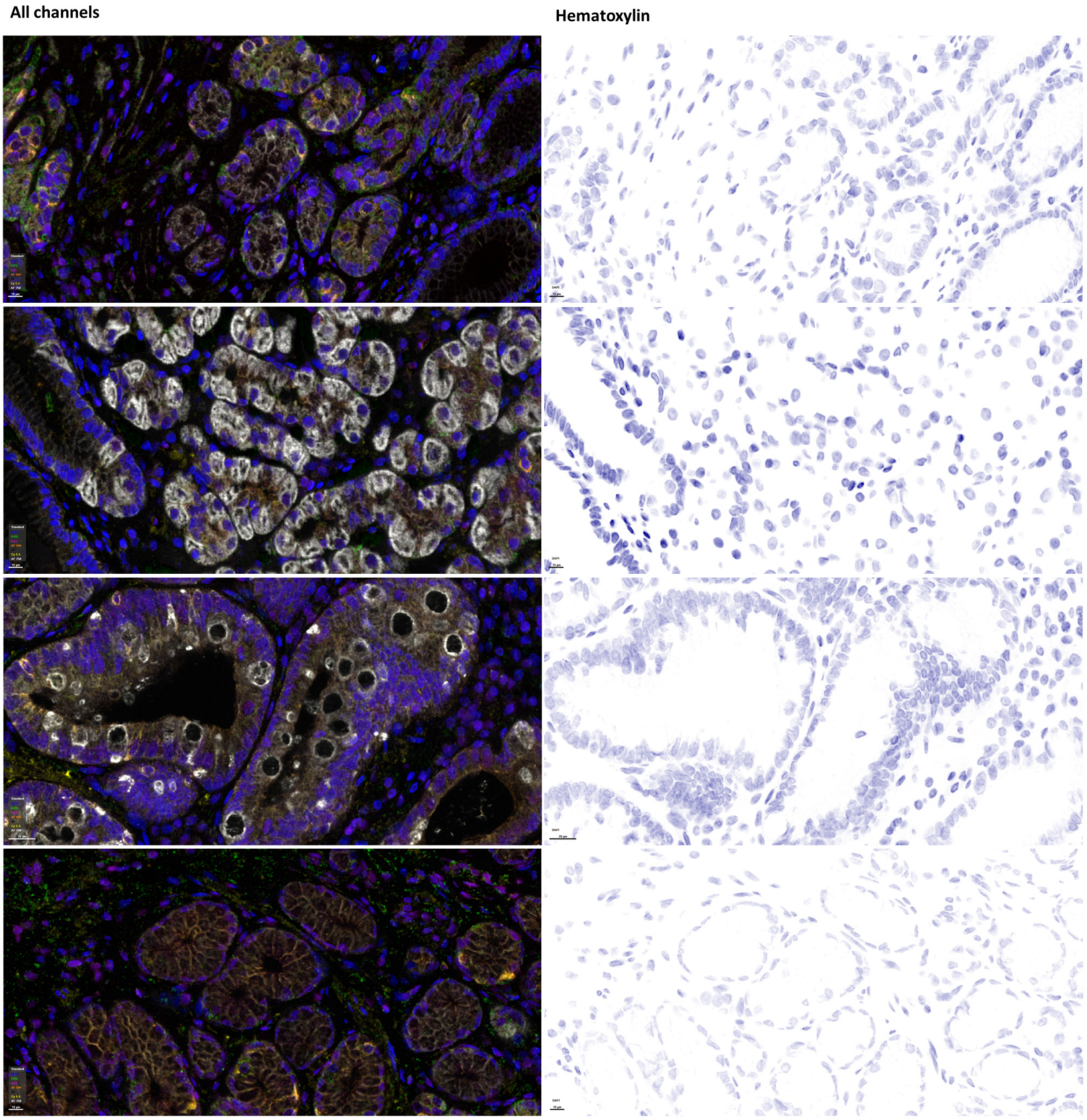

Supplementary Figure S1. Overall staining pattern in non-related tissue sections of gastric mucosa

Channel code: Nuclei (DAPI); TFF3 (Cy3); CK (FITC); TLR4 (Cy5.5); TLR5 (Alexa750); TLR9 (Alexa594). Original capture 20×, scale bar: 10 μm. The pseudo-hematoxylin staining used DAPI signal.

Supplementary Table S1. Antibody panel used for multiplexed immunofluorescence.

**Target**	**Clone**	**Dilution (μg/ml)**	**Host specie and IgG isotype**	**Assigned TSA-fluorophore**
TLR4	76B357-1	0.004	Mouse IgG2b	Cyanine5.5
TLR5	19D759.2	0.007	Mouse IgG2a	Alexa fluor 750
TLR9	26C593.2	0.002	Mouse IgG1	Alexa fluor 594
TFF3	EPR3974	0.0001	Rabbit IgG	Cyanine3
Cytokeratin	AE1/AE3	0.7	Mouse IgG1	FITC
Nuclei	none	0.0001	none	DAPI (no TSA)

TSA:Tyramide Signal Amplification.
